# Overexpression of RNF2 Is an Independent Predictor of Outcome in Patients with Urothelial Carcinoma of the Bladder Undergoing Radical Cystectomy

**DOI:** 10.1038/srep20894

**Published:** 2016-02-12

**Authors:** Xiang-Dong Li, Si-Liang Chen, Pei Dong, Jie-Wei Chen, Feng-Wei Wang, Sheng-Jie Guo, Li-Juan Jiang, Fang-Jian Zhou, Dan Xie, Zhuo-Wei Liu

**Affiliations:** 1 State Key Laboratory of Oncology in South China; Collaborative Innovation Center for Cancer Medicine, Sun Yat-sen University Cancer Center, Guangzhou, China; 2 Department of Urology, Sun Yat-sen University Cancer Center, Guangzhou, China; 3 Department of Pathology, Sun Yat-sen University Cancer Center, Guangzhou, China

## Abstract

RNF2 (ring finger protein 2) is frequently overexpressed in several types of human cancer, but the status of *RNF2* amplification and expression in urothelial carcinoma of the bladder (UCB) and its clinical/prognostic significance is unclear. In this study, immunohistochemical analysis and fluorescence *in situ* hybridization (FISH) were used to examine the expression and amplification of *RNF2* in 184 UCB patients after radical cystectomy. Overexpression of RNF2 was observed in 44.0% of UCBs and was found to significantly associate with shortened overall and cancer-specific survival (*P* < 0.001). In different subsets of UCBs, RNF2 overexpression was also identified as a prognostic indicator in patients with pT1, pT2, pN(−), and/or negative surgical margins (*P* < 0.05). Importantly, RNF2 overexpression together with pT status and surgical margin status provided significant independent prognostic parameters in multivariate analysis (*P* < 0.01). FISH results showed amplification of *RNF2* in 8/79 (10.1%) of informative UCB cases. Additionally, RNF2 overexpression was significantly associated with *RNF2* gene amplification (*P* = 0.004) and cell proliferation (*P* = 0.003). These findings suggested that overexpression of RNF2, as examined by immunohistochemical analysis, might serve as a novel prognostic biomarker and potential therapeutic target for UCB patients who undergo radical cystectomy.

Urothelial carcinoma of the bladder (UCB) is one of the most common cancers and causes of cancer-related death in the United States^1^. For patients with muscle-invasive UCB and with superficial disease at high risk of recurrence and progression, radical cystectomy plus pelvic lymphadenectomy remains the standard treatment^2^,^3^. Despite aggressive treatment with surgery and adjuvant therapy, approximately 50% of UCB patients develop metastatic/recurrent disease within 2 years^4^,^5^. Conventionally, the assignment of risk for predicting UCB patients’ recurrence and progression is mainly based on the pathologic stage, lymph node status, and positive surgical margins^6^,^7^,^8^. In clinic, however, selection of patients for multimodal treatment still remains a significant challenge^9^. Therefore, identifying specific molecular markers that determine the biological nature and behavior of UCB would be of the utmost importance for the optimization of individualized therapy.

RNF2, also called Ring1B, was first identified as an important interactor of Bmi1, which is a group II polycomb group (PcG) protein^10^. PcG proteins, which are highly expressed in many types of human cancers, exist in at least two multimeric nuclear protein complexes: polycomb repressor complex 1 (PRC1) and PRC2^11^,^12^. RNF2 is contained in the PRC1 complex, acting as an ubiquitin E3 ligase toward histone H2A at lysine 119^13^,^14^. In selective tumor types, RNF2 can promote tumor development through poly ubiquitinates tumor suppressor TP53^15^. In HeLa cells, knockdown of RNF2 could lead to an apparent morphologic change and/or a dramatic inhibition of cell proliferation^13^. A recent study reported that RNF2 was overexpressed in ductal breast carcinoma cells and involved in tumor progression^16^. These observations imply that upregulation of *RNF2* may provide a selective advantage in the pathogenesis of different human cancers. To date, however, the molecular status of *RNF2* and its potential biological role in UCB has not been elucidated. In the present study, immunohistochemical (IHC) analysis and fluorescence *in situ* hybridization (FISH) were used to examine the protein expression and amplification of the *RNF2* gene in a large cohort of UCBs; the clinicopathologic/prognostic significance of RNF2 expression in UCB patients was also assessed.

## Methods

The Ethical Committee of Sun Yat-sen University Cancer Center (Guangzhou, China) approved all the experimental methods in the current study. All experiments were done in accordance with guidelines from the Ethical Committee of Sun Yat-sen University Cancer Center.

### Patient tissue specimens

This study included 203 consecutive patients who underwent radical cystectomy plus bilateral pelvic lymphadenectomy from February 2000 to October 2009 at Sun Yat-sen University Cancer Center. One hundred eighty-four patients with primary UCB were selected; 19 patients with a history of any other type of tumor, including adenocarcinoma, squamous cell carcinoma, small-cell carcinoma, and sarcoma, were excluded. None of the patients received pelvic irradiation or systemic chemotherapy before cystectomy. The medical records of all patients were retrospectively reviewed with emphasis on overall survival (OS) and cancer-specific survival (CSS). CSS was determined from the date of surgery to the date of death from UCB or last follow-up. Formalin-fixed, paraffin-embedded (FFPE) tissues were obtained from the archives of the Department of Pathology of Sun Yat-sen University Cancer Center. Written informed consent was obtained from all patients prior to the study. The use of the clinical specimens for research purposes was approved by the Institutional Research Ethics Committee. Urologic pathologists (Drs. JW Chen and D Xie) reviewed all the histologic samples to determine the pathologic stage, according to the TNM classification criteria established by the International Union Against Cancer (6th edition, 2002). A positive surgical margin was defined as the presence of tumor at inked areas of soft tissue on the cystectomy specimen^8^. Urethral or ureteral margin status was not considered in this analysis.

### IHC analysis

IHC studies were performed using a standard streptavidin-biotin-peroxidase complex method^17^. In brief, tissue sections were deparaffinized and rehydrated. Endogenous peroxidase activity was blocked with 0.3% hydrogen peroxide for 20 min. For antigen retrieval, tissue slides were boiled in 10 mM citrate buffer (pH 6.0) in a pressure cooker for 10 min (RNF2) or microwave-treated for 10 min (Ki-67). Nonspecific binding was blocked with 10% normal rabbit serum for 20 min. The slides were incubated with anti-RNF2 (Abcam, Cambridge, MA; diluted 1:500 in phosphate buffered saline (PBS), overnight at 4 °C), and anti- Ki-67 (Abcam, Cambridge, MA; diluted 1:100 in PBS, overnight at 4 °C). All incubations were performed in a moist chamber. Subsequently, the slides were incubated with biotinylated rabbit antimouse immunoglobulin at a concentration of 1:100 for 30 min at 37 °C and then reacted with a streptavidin-peroxdase conjugate for 30 min at 37 °C and using 3′-3′ diaminobenzidine as a chromogen substrate. The nucleus was counterstained using Mayer’s hematoxylin. A negative control was obtained by replacing the primary antibody with a normal murine IgG. Known IHC-positive RNF2 staining slides of breast cancer were used as positive controls. The malignant and nonmalignant tissues were scored for RNF2 and Ki-67 by assessing the site of positive staining in the nucleus. The status of nuclear expression of RNF2 and Ki-67 was assessed by determining the percentage of positive cells stained in each tissue section. A minimum of 400 epithelial cells were counted for each case. Two independent pathologists (Drs. JW Chen and D Xie) were blinded to the clinicopathologic information and performed the scorings. Inter observer disagreements (which occurred in about 6% of the total informative cases) were reviewed a second time, and both pathologists subsequently rendered a final judgment.

### FISH

Two-color FISH was applied to the sections of FFPE UCB tissues using spectrum red-labeled bacterial artificial chromosome clone (CH17-111I15) containing the RNF2 gene; a chromosome 1 centromere probe labeled by spectrum green (Vysis, Downers Grove, IL) was used as internal control. The FISH reaction was performed as described previously^18^ with slight modification. Briefly, the deparaffinized tissue section was treated with proteinase K (400 μg/ml) at 37 °C for 45 min, followed by denaturing in 70% formamide, 2× standard saline citrate (SSC) at 75 °C for 8 min. 50 ng of each probe were mixed in a 20-μl-hybridization mixture (containing 55% formamide, 2× SSC, and 2 μg human Cot1 DNA), denatured at 75 °C for 6 minutes and then hybridized to the denatured FFPE section at 37 °C for 24 hours. After washing, the section was counterstained with 1 μg/ml DAPI (4’,6-diamidino-2-phenylindole) in an anti-fade solution and examined with a Zeiss Axiophot microscope equipped with a triple-band pass filter. FISH signals from 300 cells in each sample were counted. The criteria for gene amplification of RNF2 were defined as the presence (in ≥20% of tumor cells) of either 6 (or more) gene signals or more than 3 times as many gene signals as reference signals.

### Statistical analysis

Statistical analysis was performed with SPSS software (SPSS version 17.0, SPSS Inc.). The chi-square test was used to determine the association of RNF2 protein expression with the UCB patient’s clinicopathologic features and the correlations between molecular features detected with each. For survival analysis, we analyzed all UCB patients by the Kaplan-Meier method. The log-rank test was used to compare different survival curves. Multivariate survival analysis using the Cox regression model was performed on all parameters that were found to be significant on univariate analysis. *P* values < 0.05 were considered statistically significant.

## Results

### RNF2 expression in UCB and normal bladder tissues

**Figure 1:**
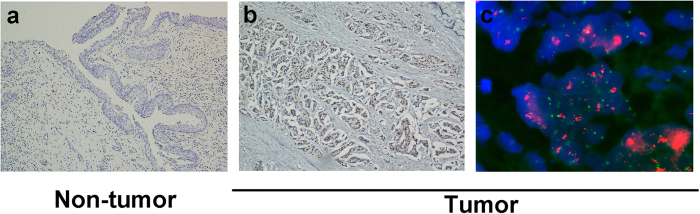
Immunohistochemical staining of RNF2 and fluorescence *in situ* hybridization (FISH) of RNF2 in human bladder tissues. (**a**) A normal bladder mucosa showed normal expression of RNF2 protein with a negative staining of RNF2 in nuclei of all normal bladder epithelial cells. Original magnification ×100. (**b**) A UCB (case 115) showing overexpression of RNF2, in which more than 90% of tumor cells had positive staining of RNF2 in nuclei. Original magnification ×100. (**c**) Amplification of the *RNF2*gene was observed by FISH in the same UCB case (case 115), in which *RNF2* genes signals (red) were detected at least 3 times more than signals on chromosome 1 centromere (green). Original magnification ×1000.

Positive expression of RNF2 by IHC analysis in UCB cells and in normal bladder mucosa cells was primarily a nuclear pattern (Fig. 1a,b). Because the expression of RNF2 in each of the normal bladder mucosa was negative or no more than 10% of the urothelium with positive staining (Fig. 1a), overexpression of RNF2 was scored only when more than 10% of the tumor cell nuclei were positively stained (Fig. 1b). Using these criteria, the overexpression of RNF2 was examined in 81/184 (44.0%) of the UCBs. In addition, we evaluated a potential association between RNF2 expression and tumor clinicopathologic features in UCBs. The results showed that no significant association was determined between expression of RNF2 and the clinicopathologic features of the UCB cohort, such as patient age, sex, smoking history, surgical margins status, pT status, pN status, and multiplicity (*P* > 0.05; Table 1).

### Relationship between clinicopathologic variables, RNF2 expression, and UCB patient survival

**Figure 2:**
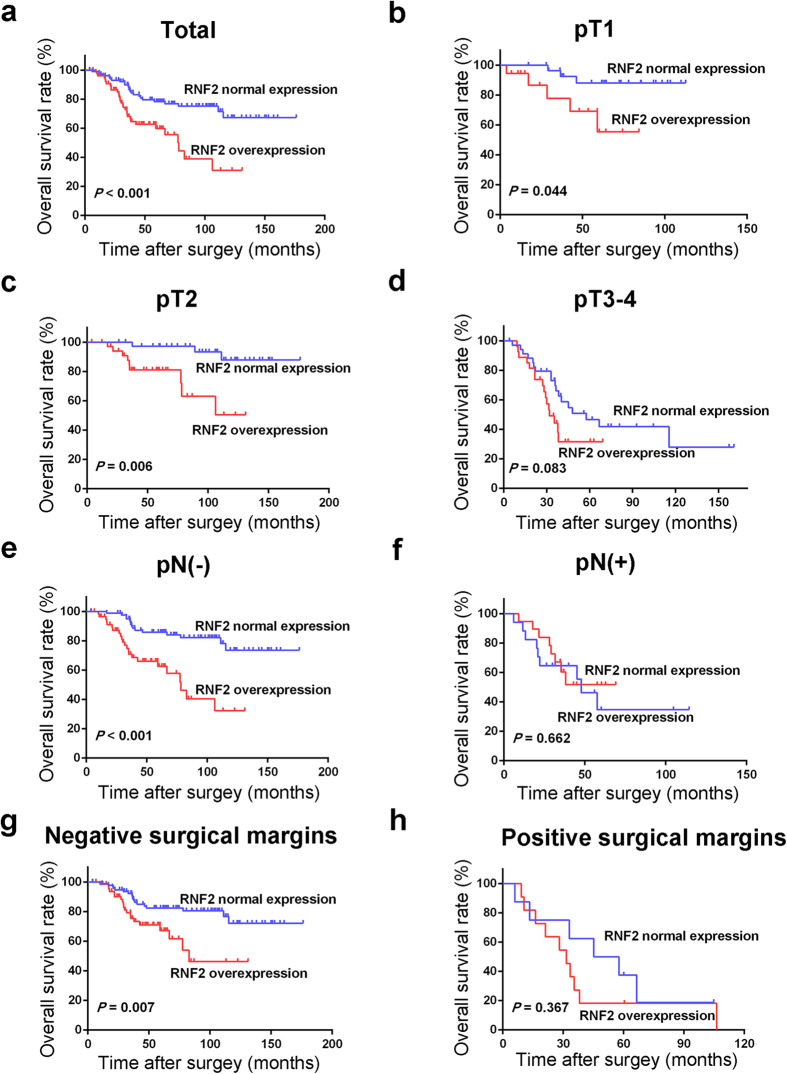
Overall survival analysis of RNF2 expression in total cohort and different subsets of UCB patients. (**a**) Total cohort; (**b**) pT1; (**c**) pT2; (**d**) pT3/4; (**e**) pN(−); (**f**) pN(+); (**g**) Negative surgical margins; (**h**) Positive surgical margins.

**Figure 3:**
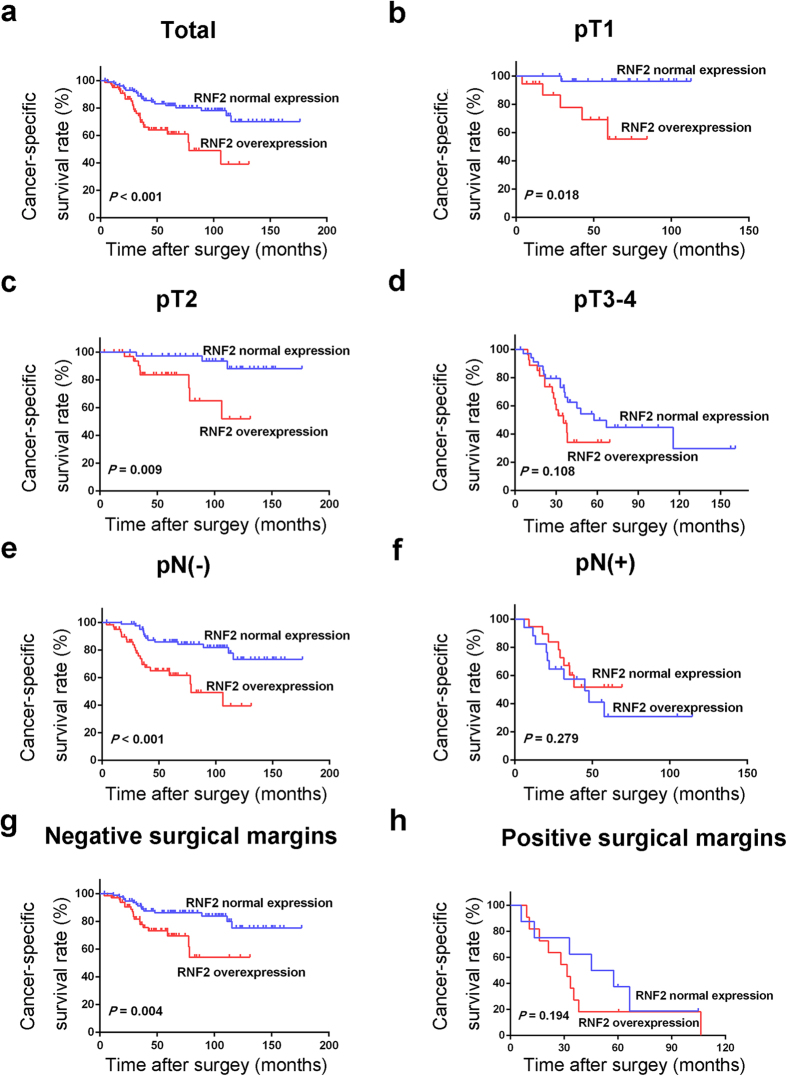
Cancer-specific survival analysis of RNF2 expression in total cohort and different subsets of UCB patients. (**a**) Total cohort; (**b**) pT1; (**c**) pT2; (**d**) pT3/4; (**e**) pN(−); (**f**) pN(+); (**g**) Negative surgical margins; (**h**) Positive surgical margins.

In our cohort, the median follow-up time of UCB patients was 69 months (range, 6–176 months). In univariate analysis, overexpression of RNF2 was evaluated to correlate closely with poor patient OS and CSS for the whole UCB cohort and could further stratify patient survival in pT1, pT2, pN(−), and negative surgical margin (Table 2, Figs 2 and 3). Multivariate analysis demonstrated that RNF2 expression, as well as pT status and surgical margin status, were independent predictors of OS and CSS for the whole UCB cohort (Table 3).

### Correlation between RNF2 expression and cell proliferation in UCBs

**Figure 4:**
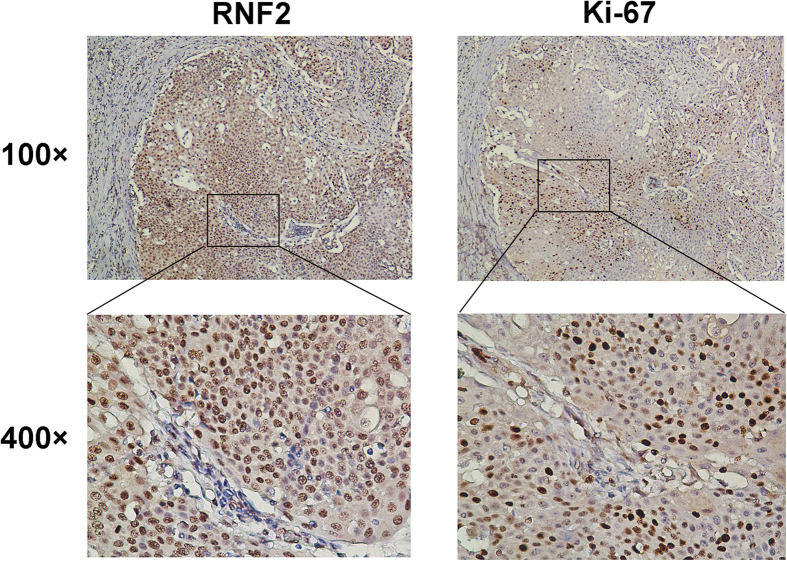
RNF2 levels are positively correlated with Ki-67 expression in primary UCB specimens. Representative image shows that overexpression of RNF2 and high-level expression of Ki-67 was examined by IHC in a UCB case (case 36).

The expression level of Ki-67 was assessed as a labeling index (LI) (i.e., as the percentage of Ki-67-positive cells in each tumor). In our UCB cohorts, since the median LI value of Ki-67 for all 184 UCB tumor samples was 28.2%, the median value of 28.2% was used as a cutoff value to define low LI of Ki-67 (LI < 28.2%) and high LI of Ki-67 (LI ≥ 28.2%). In our IHC samples, RNF2 and Ki-67 were detected simultaneously (Fig. 4). Correlation analysis demonstrated that the frequency of cases with a high LI of Ki-67 was significantly greater in tumors with overexpression of RNF2 (58/81 cases, 71.6%) than in tumors with normal expression of RNF2 (34/103, 33.0%; chi-square test, *P* = 0.003).

### Amplification of *RNF2* in UCBs

In our FISH study, FISH analysis was informative in 79/184 (42.9%) of the UCBs. Samples without FISH signal, or samples with weak target signals or samples with a strong signal background, comprised the non-informative cases. FISH results showed that the amplification of *RNF2* was determined in 8/79 (10.1%) of informative UCB cases; in each of the 7 cases with *RNF2* amplification, overexpression of RNF2 was observed (Fig. 1c). In the remaining 71 informative cancers without *RNF2* amplification, 46 (64.8%) cases showed normal expression of RNF2, while 25 (35.2%) cases showed overexpression of RNF2 (Table 4).

## Discussion

Amplification of 1q is one of the most frequent chromosomal aberrations in human UCB^19^,^20^ and in several other types of cancer, including lung^21^, breast^22^, colorectal^23^, and hepatocellular^24^. This suggests that human chromosome 1q contains oncogenes related to tumorigenesis and/or progression of human cancers. In addition to genomic alterations, epigenetic changes have been associated with tumor development. Recent evidence showed that PcG proteins are highly expressed in various kinds of human cancers and function as transcriptional repressors. As a core member of the PRC1 complex, RNF2 from chromosome 1q25.3 has been previously found to have an oncogenic role in selective cancers^15^,^16^. This prompted us to investigate whether the abnormalities of RNF2 are involved in the pathogenesis of UCB.

In our study, we observed that, when examined by IHC analysis, expression of RNF2 in all normal bladder tissue specimens was absent or at low level. Levels of RNF2 expression were found to be upregulated in our UCB specimens, which suggests that RNF2 potentially plays an important role in the tumorigenesis of UCB. Although no significant association was observed between RNF2 expression and any of the UCB patients’ clinicopathologic features, including tumor grade and clinical stage, overexpression of RNF2 was a strong and independent predictor of short OS and CSS, as evidenced by Kaplan-Meier curves and multivariate Cox proportional hazards regression analysis. Furthermore, stratified survival analysis of UCB histopathological grade and/or pT stage showed that expression of RNF2 was closely associated to the OS and CSS of different subsets of UCB patients. Interestingly, we found that the prognostic significance of RNF2 overexpression was more prominent in patients with either localized pathologic tumor stage or no nodal involvement. Conversely, overexpression of RNF2 did not significantly predict OS and CSS in patients with advanced pathologic stage and/or nodal involvement. Similarly predictive patterns were also observed in patients with or without positive surgical margins. Thus, these findings suggest that the predictive effect of RNF2 overexpression on UCB oncologic outcomes is clinically significant in patients with pathologically localized tumors.

Previous studies demonstrated that RNF2 was frequently up-regulated in many types of human cancers and promoted tumor cell proliferation through negative regulation of p53^15^,^25^. In fact, p53 is crucial for keeping urothelial growth in check and the urothelial genome stable^26^. UCB patients with a down-regulated level of p53 have a much higher risk of disease progression and poorer prognosis than patients who without^27^,^28^. These data suggest that RNF2 may provide a selective advantage in the tumorigenesis of UCBs. Although we have shown overexpression of RNF2 in several of our UCB tumors, and a positive association between the expression levels of RNF2 and that of Ki-67 (an important marker for cell proliferation), the precise signaling pathway involved in these processes remains to be determined. Nevertheless, our results suggest a potentially important role for *RNF2* in the control of UCB cell proliferation, an activity that might be responsible, at least in part, for UCB tumorigenesis and/or progression.

It is well established that overexpression of an oncogene in human cancers is often caused by gene amplification^29^. To assess if the overexpression of RNF2 in UCBs was caused by gene amplification, we examined the amplification status of *RNF2* by FISH. In our 79 informative UCB cases examined simultaneously by both IHC analysis and FISH, overexpression of RNF2 was detected in 87.5% (7/8) of UCBs that had *RNF2* amplification. However, amplification of *RNF2* was not observed in 25 other UCBs with overexpression of RNF2. These results indicate that overexpression of RNF2 was associated with *RNF2* gene amplification, but it did not always coincide, suggesting that molecular mechanisms other than gene amplification might play a more critical role in the regulation of RNF2 expression in UCBs.

Our study did have some limitations. First, it was a retrospective study, and our data were obtained from a single tertiary center. Thus, future community-based prospective studies or studies from multiple centers are required for external validation. Second, the informative cases in our FISH study represented only 42.9% of our cohort; this might have biased our data. Third, our cohort specimens were all from cystectomy; future studies will need to focus on the relationship between RNF2 expression and low-grade UCBs.

In summary, our results provide some evidence that overexpression of RNF2 plays a role in the prognosis of UCB and might serve as a novel prognostic marker and potential therapeutic target for UCB patients.

## Additional Information

**How to cite this article**: Li, X.-D. *et al.* Overexpression of RNF2 Is an Independent Predictor of Outcome in Patients with Urothelial Carcinoma of the Bladder Undergoing Radical Cystectomy. *Sci. Rep.* **6**, 20894; doi: 10.1038/srep20894 (2016).

## Figures and Tables

**Table 1:** Association between RNF2 expression and tumor clinicopathologic features in 184 UCB cases.

Variable	All cases (n = 184)	RNF2 expression (%)		*P*^a^
		Normal expression (n = 103)	Overexpression (n = 81)	
Age (years)				0.743
≤60	102	56(54.9)	46(46.1)	
>60	82	47(58.5)	35(41.5)	
Sex				0.885
Men	162	91(54.5)	71(45.5)	
Women	22	12(56.2)	10(43.8)	
Tumor multiplicity				0.330
Unifocal	70	36(51.4)	34(48.6)	
Multifocal	114	67(58.8)	47(41.2)	
Smoking history				0.663
No	83	45(54.2)	38(45.8)	
Yes	101	58(57.4)	43(42.6)	
Surgical margins				0.188
Negative	165	96(58.1)	69(41.9)	
Positive	19	8(42.1)	11(57.9)	
pT status				0.277
pT1	48	30(62.5)	18(37.5)	
pT2	74	38(51.4)	36(48.6)	
pT3	41	26(63.4)	15(36.6)	
pT4	21	9(42.9)	12(57.1)	
pN status				0.238
pN−	148	86(58.1)	62(41.9)	
pN+	36	17(47.2)	19(52.8)	

^a^chi-square test.

Abbreviation: RNF2, ring finger protein 2. UCB, urothelial carcinoma of the bladder.

**Table 2:** Prognostic value of RNF2 expression in 184 UCB cases.

RNF2 expression	Cases	Overall survival			Cancer-specific survival		
		Hazard ratios	95% confidence interval	*P*^a^	Hazard ratios	95% confidence interval	*P*^a^
Total							
Normal expression	103	1			1		
Overexpression	81	2.644	1.533–4.562	<0.001	2.945	1.630–5.323	<0.001
pT status							
pT1							
Normal expression	30	1			1		
Overexpression	18	4.403	1.044–18.569	0.044	13.373	1.548–115.517	0.018
pT2							
Normal expression	38	1			1		
Overexpression	36	6.687	1.736–25.758	0.006	6.150	1.561–24.236	0.009
pT3-T4							
Normal expression	35	1			1		
Overexpression	27	1.847	0.916–3.724	0.083	1.811	0.878–3.736	0.108
pN status							
pN−							
Normal expression	86	1			1		
Overexpression	62	3.825	1.968–7.434	<0.001	10.173	4.218–24.540	<0.001
pN+							
Normal expression	17	1			1		
Overexpression	19	0.807	0.309–2.109	0.662	0.592	0.229–1.530	0.279
Surgical margins							
negative							
Normal expression	96	1			1		
Overexpression	69	2.446	1.274–4.695	0.007	2.850	1.393–5.831	0.004
positive							
Normal expression	8	1			1		
Overexpression	11	1.627	0.565–4.683	0.367	2.130	0.680–6.670	0.194

^a^chi-square test.

Abbreviation: RNF2, ring finger protein 2. UCB, urothelial carcinoma of the bladder.

**Table 3:** Multivariate Cox regression analysis for overall survival of UCB patients.

Factor	Overall survival			Cancer-specific survival		
	Hazard ratio	95% confidence interval	*P*	Hazard ratio	95% confidence interval	*P*
RNF2 expression^a^	2.397	1.339–4.292	0.003	2.560	1.388–4.723	0.003
pT status^b^	2.735	1.582–4.729	<0.001	2.783	1.580–5.225	0.001
pN status^c^	0.680	0.318–1.457	0.322	0.838	0.381–1.839	0.659
Surgical margins^d^	3.316	1.683–6.534	0.001	3.351	1.677–6.694	0.001

^a^Normal expression vs. overexpression; ^b^pT1 vs.T2 vs. pT3-pT4; ^c^pN- vs. pN+.

^d^negative vs. positive.

Abbreviation: RNF2, ring finger protein 2. UCB, urothelial carcinoma of the bladder.

**Table 4:** Association between *RNF2* expression and amplification in UCBs.

*RNF2* gene	Informative cases	RNF2 expression		*P*^a^
		Normal expression	Overexpression	
No amplification	71	46 (64.8)	25 (35.2)	0.004
Amplification	8	1 (12.5)	7 (87.5)	

^a^chi-square test.

Abbreviation: RNF2, ring finger protein 2. UCB, urothelial carcinoma of the bladder.

## References

[b1] Jacobs B. L., Lee C. T. & Montie J. E. Bladder cancer in 2010: how far have we come? CA: a cancer journal for clinicians 60, 244–272, doi: 10.3322/caac.20077 (2010). 20566675

[b2] Stenzl A. *et al.* Treatment of muscle-invasive and metastatic bladder cancer: update of the EAU guidelines. European urology 59, 1009–1018, doi: 10.1016/j.eururo.2011.03.023 (2011). 21454009

[b3] Hautmann R. E. *et al.* Urinary diversion. Urology 69, 17–49, doi: 10.1016/j.urology.2006.05.058 (2007). 17280907

[b4] Sternberg C. N. *et al.* ICUD-EAU International Consultation on Bladder Cancer 2012: Chemotherapy for urothelial carcinoma-neoadjuvant and adjuvant settings. European urology 63, 58–66, doi: 10.1016/j.eururo.2012.08.010 (2013). 22917984

[b5] Zhang Z. L. *et al.* Radical cystectomy for bladder cancer: oncologic outcome in 271 Chinese patients. Chinese journal of cancer 33, 165–171, doi: 10.5732/cjc.012.10312 (2014). 23958053 PMC3966145

[b6] Stein J. P. *et al.* Radical cystectomy in the treatment of invasive bladder cancer: long-term results in 1,054 patients. Journal of clinical oncology: official journal of the American Society of Clinical Oncology 19, 666–675 (2001). 11157016 10.1200/JCO.2001.19.3.666

[b7] Herr H. W. *et al.* Surgical factors influence bladder cancer outcomes: a cooperative group report. Journal of clinical oncology : official journal of the American Society of Clinical Oncology 22, 2781–2789, doi: 10.1200/jco.2004.11.024 (2004). 15199091

[b8] Novara G. *et al.* Soft tissue surgical margin status is a powerful predictor of outcomes after radical cystectomy: a multicenter study of more than 4,400 patients. The Journal of urology 183, 2165–2170, doi: 10.1016/j.juro.2010.02.021 (2010). 20399473

[b9] Prasad S. M., Decastro G. J. & Steinberg G. D. Urothelial carcinoma of the bladder: definition, treatment and future efforts. Nature reviews. Urology 8, 631–642, doi: 10.1038/nrurol.2011.144 (2011). 21989305

[b10] Levine S. S. *et al.* The core of the polycomb repressive complex is compositionally and functionally conserved in flies and humans. Molecular and cellular biology 22, 6070–6078 (2002). 12167701 10.1128/MCB.22.17.6070-6078.2002 PMC134016

[b11] Otte A. P. & Kwaks T. H. Gene repression by Polycomb group protein complexes: a distinct complex for every occasion? Current opinion in genetics & development 13, 448–454 (2003). 14550408 10.1016/s0959-437x(03)00108-4

[b12] Scelfo A., Piunti A. & Pasini D. The controversial role of the Polycomb group proteins in transcription and cancer: how much do we not understand Polycomb proteins? The FEBS journal 282, 1703–1722, doi: 10.1111/febs.13112 (2015). 25315766

[b13] Wang H. *et al.* Role of histone H2A ubiquitination in Polycomb silencing. Nature 431, 873–878, doi: 10.1038/nature02985 (2004). 15386022

[b14] Buchwald G. *et al.* Structure and E3-ligase activity of the Ring-Ring complex of polycomb proteins Bmi1 and Ring1b. The EMBO journal 25, 2465–2474, doi: 10.1038/sj.emboj.7601144 (2006). 16710298 PMC1478191

[b15] Su W. J. *et al.* RNF2/Ring1b negatively regulates p53 expression in selective cancer cell types to promote tumor development. Proceedings of the National Academy of Sciences of the United States of America 110, 1720–1725, doi: 10.1073/pnas.1211604110 (2013). 23319651 PMC3562849

[b16] Bosch A. *et al.* The Polycomb group protein RING1B is overexpressed in ductal breast carcinoma and is required to sustain FAK steady state levels in breast cancer epithelial cells. Oncotarget 5, 2065–2076 (2014). 24742605 10.18632/oncotarget.1779 PMC4039145

[b17] Cai M. Y. *et al.* EZH2 protein: a promising immunomarker for the detection of hepatocellular carcinomas in liver needle biopsies. Gut 60, 967–976, doi: 10.1136/gut.2010.231993 (2011). 21330577

[b18] Xie D. *et al.* Correlation of AIB1 overexpression with advanced clinical stage of human colorectal carcinoma. Human pathology 36, 777–783, doi: 10.1016/j.humpath.2005.05.007 (2005). 16084947

[b19] Hopman A. H. *et al.* Numerical chromosome 1, 7, 9, and 11 aberrations in bladder cancer detected by *in situ* hybridization. Cancer research 51, 644–651 (1991). 1985781

[b20] Poddighe P. J., Ramaekers F. C., Smeets A. W., Vooijs G. P. & Hopman A. H. Structural chromosome 1 aberrations in transitional cell carcinoma of the bladder: interphase cytogenetics combining a centromeric, telomeric, and library DNA probe. Cancer research 52, 4929–4934 (1992). 1516049

[b21] Ma J. *et al.* Gain of 1q25-32, 12q23-24.3, and 17q12-22 facilitates tumorigenesis and progression of human squamous cell lung cancer. The Journal of pathology 210, 205–213, doi: 10.1002/path.2050 (2006). 16927450

[b22] Mesquita B. *et al.* Frequent copy number gains at 1q21 and 1q32 are associated with overexpression of the ETS transcription factors ETV3 and ELF3 in breast cancer irrespective of molecular subtypes. Breast cancer research and treatment 138, 37–45, doi: 10.1007/s10549-013-2408-2 (2013). 23329352

[b23] Kassouf W. *et al.* Lymph node density is superior to TNM nodal status in predicting disease-specific survival after radical cystectomy for bladder cancer: analysis of pooled data from MDACC and MSKCC. Journal of clinical oncology: official journal of the American Society of Clinical Oncology 26, 121–126, doi: 10.1200/jco.2007.12.9247 (2008). 18165646

[b24] Ma N. F. *et al.* Isolation and characterization of a novel oncogene, amplified in liver cancer 1, within a commonly amplified region at 1q21 in hepatocellular carcinoma. Hepatology (Baltimore, Md.) 47, 503–510, doi: 10.1002/hep.22072 (2008). 18023026

[b25] Wen W. *et al.* Knockdown of RNF2 induces apoptosis by regulating MDM2 and p53 stability. Oncogene 33, 421–428, doi: 10.1038/onc.2012.605 (2014). 23318437 PMC3920452

[b26] Wu X. R. Urothelial tumorigenesis: a tale of divergent pathways. Nature reviews. Cancer 5, 713–725, doi: 10.1038/nrc1697 (2005). 16110317

[b27] Hartmann A. *et al.* Occurrence of chromosome 9 and p53 alterations in multifocal dysplasia and carcinoma *in situ* of human urinary bladder. Cancer research 62, 809–818 (2002). 11830537

[b28] Esrig D. *et al.* Accumulation of nuclear p53 and tumor progression in bladder cancer. The New England journal of medicine 331, 1259–1264, doi: 10.1056/nejm199411103311903 (1994). 7935683

[b29] Stark G. R., Debatisse M., Giulotto E. & Wahl G. M. Recent progress in understanding mechanisms of mammalian DNA amplification. Cell 57, 901–908 (1989). 2661014 10.1016/0092-8674(89)90328-0

